# Analysis of Antibiotic Resistance Patterns and Distribution of *Staphylococcus aureus* in the Middle East: A Systematic Review and Meta‐Analysis

**DOI:** 10.1155/cjid/2830917

**Published:** 2026-09-26

**Authors:** Sarina Alidadpour, Reza Pakzad, Mohammad Hossein Ekvan, Muhammad Ibrahim Getso, Gita Alizadeh, Wrya Parwaie, Omid Raiesi

**Affiliations:** ^1^ Student Research Committee, Ilam University of Medical Sciences, Ilam, Iran, medilam.ac.ir; ^2^ Department of Epidemiology, School of Health, Ilam University of Medical Sciences, Ilam, Iran, medilam.ac.ir; ^3^ Department of Medical Microbiology and Parasitology, College of Health Sciences, Bayero University, Kano PMB, 3011, Nigeria, buk.edu.ng; ^4^ Department of Parasitology and Mycology, School of Medicine, Iran University of Medical Sciences, Tehran, Iran, iums.ac.ir; ^5^ Department of Medical Physics, Faculty of Paramedical Sciences, Ilam University of Medical Sciences, Ilam, Iran, medilam.ac.ir; ^6^ Department of Parasitology, School of Allied Medical Sciences, Ilam University of Medical Sciences, Ilam, Iran, medilam.ac.ir; ^7^ Zoonotic Diseases Research Center, Ilam University of Medical Sciences, Ilam, Iran, medilam.ac.ir

**Keywords:** antibiotic resistance, meta-analysis, Middle East, *Staphylococcus aureus*, systematic review

## Abstract

**Background:**

Antimicrobial resistance (AMR) is one of the most critical health concerns of the 21^st^ century, leading to increased mortality, prolonged hospital stays for patients, and higher healthcare costs. Despite urgency of AMR issue, comprehensive and comparative data on AMR in the Middle East region remain limited. This study aimed to systematically review and synthesize country‐level data on MRSA prevalence and resistance rates to seven commonly reported antibiotics, with the goal of identifying knowledge gaps and supporting evidence‐based antimicrobial stewardship policies and clinical decision‐making.

**Methods:**

A comprehensive literature search was conducted across four electronic databases: PubMed, Web of Science (WOS), Scopus, and Google Scholar. The search included articles published from January 2015 to September 24, 2024. Search terms covered *Staphylococcus aureus,* AMR, and Middle Eastern countries. Ultimately, 217 studies with a combined sample size exceeding 32,090 were included in the analysis.

**Results:**

In Iran, the highest and lowest pooled resistance rates were for penicillin G and vancomycin, respectively. For MRSA, the highest pooled resistance was observed in Iraq and the lowest in Turkey. Vancomycin pooled resistance was also the highest in Iraq. Gentamicin resistance was highest in Yemen and lowest in Cyprus and Kuwait. The highest pooled resistance rates for gentamicin, cotrimoxazole, and ciprofloxacin were all observed in Yemen.

**Conclusions:**

Factors contributing to high AMR rates include ineffective antibiotic stewardship, self‐medication, prescription errors, inappropriate dosing, and excessive antibiotic use in agriculture. The findings highlight the urgent need for stricter antibiotic regulations, improved stewardship, and public awareness across the Middle East.

## 1. Background

Infectious diseases are the second leading cause of human mortality worldwide [1]. *Staphylococcus aureus* (*S. aureus*), a Gram‐positive bacterium, is a major contributor to morbidity and mortality, causing conditions such as endocarditis, bacteremia, skin and soft tissue infections, osteomyelitis, and lethal pneumonia [2, 3]. Based on antibiotic sensitivity, *S. aureus* can be divided into methicillin‐sensitive *Staphylococcus aureus* (MSSA) and methicillin‐resistant *Staphylococcus aureus* (MRSA) strains. Over recent decades, bacterial evolution and widespread antibiotic misuse have driven a global increase in MRSA infections, complicating clinical case management [1]. Vancomycin has long been the most effective antibiotic agent against MRSA infections. However, in 1996 the first vancomycin‐resistant *S. aureus* (VRSA) strains was isolated in Japan, and subsequent reports from France, USA, Korea, South Africa, and Brazil confirmed VRSA strain emergence as a global problem [4]. Antibiotic resistance problem threatens human existence. Antibiotic resistance is a global health threat. Frequent use of vancomycin as first‐line therapy has contributed to the emergence of vancomycin‐intermediate *Staphylococcus aureus* (VISA) and vancomycin‐resistant *Staphylococcus aureus* (VRSA) strains [5]. Current treatment options for β‐lactam‐resistant *S. aureus* include glycopeptides, cyclic lipopeptides, cephalosporins, and oxazolidinones, represented by vancomycin, daptomycin, ceftaroline, and linezolid, respectively [5]. Recent years have witnessed the emergence of multidrug‐resistant *Staphylococcus aureus* strains with reduced susceptibility to last‐line antimicrobial agents. Although vancomycin remains the cornerstone of therapy for many MRSA infections, increasing reports of VISA and VRSA have raised concerns regarding future treatment options. Consequently, newer antistaphylococcal agents, including ceftaroline, ceftobiprole, dalbavancin, tedizolid, and delafloxacin, have been introduced in several healthcare settings as alternative therapeutic options. Continuous surveillance of resistance patterns is therefore essential to preserve the effectiveness of both existing and newly developed antimicrobial agents [6–8]. Antibiotic resistance is a global health crisis threatening the golden age of antibiotic therapy. High resistance rates complicate empirical therapy, severely restrict antibiotic choices, and occur alongside a dwindling pipeline of new antibiotics. Although resistance cannot be entirely prevented, its development and spread can be mitigated through appropriate interventions [9]. Epidemiological surveillance is a critical approach to understanding and addressing antibiotic resistance [10]. Surveillance involves the collecting and analyzing of data for the detection and monitoring of threats to public health. Antimicrobial surveillance involves collecting and analyzing antibiotic susceptibility results from clinical microbiology laboratories. When combined with demographic and clinical data for the patient populations, these data provide insight into resistance patterns and inform rational interventions that can be used to reduce the burden of resistance [11]. There is a paucity of consolidated and comparative data on *Staphylococcus aureus* resistance patterns across the Middle East. Therefore, this study aimed to systematically review and analyze available surveillance data to identify trends, compare resistance rates, and evaluate geographic variations. Our purpose is to provide information that can inform regional antibiotic policies and clinical decision‐making. A meta‐analysis was conducted to assess the regional patterns, highlight successful strategies, and uncover gaps in surveillance. Based on the observed resistance trends, the findings may serve as a useful tool to optimize empirical therapy. To our knowledge, this is the first meta‐analysis to compare *S. aureus* resistance across the Middle East, addressing a critical gap in evidence and guiding both public health planning and antimicrobial stewardship.

## 2. Materials and Methods

### 2.1. Protocol Registration

This systematic review and meta‐analysis was conducted according to PRISMA 2020 guidelines, and the study protocol was registered in PROSPERO (Registration No: CRD42024596444).

### 2.2. Search Strategy

A comprehensive literature search was conducted across four electronic databases: PubMed, Web of Science (WOS), Scopus, and Google Scholar. The search included articles published from January 2015 to September 24, 2024. Search terms combined Medical Subject Headings (MeSH) and keywords related to *Staphylococcus aureus*, antibiotic resistance (including terms such as “drug resistance,” “drug susceptibility,” and “antimicrobial resistance”), and geographic locations within the Middle East, including Bahrain, Iran, Iraq, Israel, Jordan, Kuwait, Lebanon, Oman, Qatar, Saudi Arabia, Syria, Turkey, United Arab Emirates, Yemen, Palestine, Cyprus, Egypt, Afghanistan, and Pakistan. The final search was conducted on September 24, 2024. Database‐specific search strategies were developed using a combination of MeSH terms and free‐text keywords. Detailed search strings, Boolean operators, and database‐specific search strategies are provided in Supporting Table S1. For the purposes of this review, the geographical scope was based on the broader Middle East and neighboring countries frequently included in regional antimicrobial resistance surveillance reports and public health assessments. Therefore, Afghanistan and Pakistan were included because of their epidemiological, healthcare, and antimicrobial use similarities with several Middle Eastern countries and their frequent inclusion in regional infectious disease surveillance frameworks.

### 2.3. Inclusion and Exclusion Criteria

Studies were included if they met the following criteria:

Cross‐sectional or surveillance studies, studies reporting antibiotic resistance rates of *Staphylococcus aureus* isolates within Middle Eastern countries, published in English, and provided quantitative data on antibiotic susceptibility by specific antibiotic type.

Exclusion criteria were studies on nonhuman samples or environmental sources, studies focusing on specific strains of *S. aureus* only, studies with fewer than 10 isolates, and studies without accessible full text or insufficient data for resistance rate extraction were excluded. This criterion was applied to studies restricted to selected molecular lineages, clonal complexes, or highly specific subpopulations of *S. aureus*, as such studies may not reflect the overall resistance profile of circulating clinical isolates within a country. The objective of the present review was to estimate resistance patterns at the population level; therefore, studies limited to narrowly defined strain groups were excluded to improve comparability and representativeness across countries.

### 2.4. Study Selection

Screening was performed in three phases by two independent reviewers. Disagreements were resolved through discussion or consultation with a third independent reviewer.

### 2.5. Data Extraction

Date extraction included publication year, country, sample size, types of antibiotics tested, and reported resistance rates from each included study. Two independent reviewers performed the data extraction, using a data collection form.

### 2.6. Quality Assessment

The Joanna Briggs Institute (JBI) checklist was used to assess bias risk and study quality. Studies with high bias risk were noted but not excluded.

### 2.7. Data Analysis

Statistical analyses were performed using STATA Version 18 (StataCorp LLC, College Station, TX, USA). Pooled resistance rates were estimated using a random‐effects model. Because resistance outcomes were analyzed as proportions, the Freeman–Tukey double arcsine transformation was applied to stabilize variances and allow the inclusion of studies reporting resistance rates of 0% or 100%. The pooled estimates and corresponding 95% confidence intervals (CIs) were subsequently back‐transformed to the original proportion scale for interpretation. Between‐study heterogeneity was assessed using Cochran’s Q test and quantified with the I^2^ statistic. Given the substantial heterogeneity across studies, subgroup analyses were performed according to country. Univariate meta‐regression analyses were conducted to investigate the potential influence of publication year, sample size, and country on the observed heterogeneity. Publication bias was assessed using funnel plot inspection and Egger’s regression test. Sensitivity analyses were performed using a leave‐one‐out approach, whereby each study was sequentially omitted and the pooled estimate was recalculated to evaluate the robustness of the findings.

### 2.8. Publication Bias Assessment

Publication bias was assessed using funnel plot inspection and Egger’s regression test. Outcomes with a sufficient number of studies (> 10 studies) were included in the analysis. Funnel plot asymmetry and Egger’s test *p* values were used to evaluate potential small‐study effects.

### 2.9. Sensitivity Analysis

Sensitivity analyses were conducted using a leave‐one‐out approach, whereby each study was sequentially omitted and the pooled estimate was recalculated. This procedure was used to evaluate the influence of individual studies on the overall resistance estimates and to assess the robustness of the findings.

## 3. Results

**FIGURE 1:**
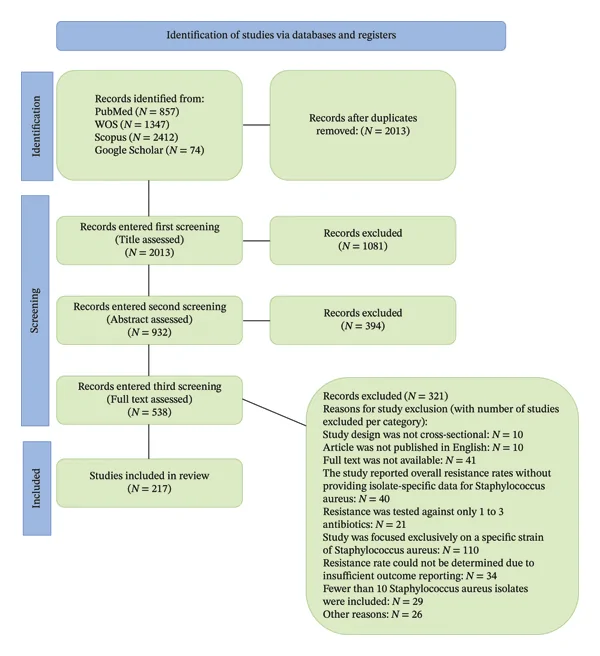
Flowchart of systematic review and meta‐analysis.

A total of 4690 studies were initially identified through database searches. After removing duplicates, 2013 articles remained for screening, which was conducted in three stages. In the first stage, 1475 studies were excluded based on title and abstract review. In the second stage, full texts of the remaining 538 studies were assessed, leading to exclusion of 321 studies. Ultimately, 217 studies with a combined sample size exceeding 32,090 were included in the analysis. The detailed selection process is illustrated in Figure 1. Among the included studies, Iran contributed the highest number (*n* = 111), followed by Iraq (*n* = 24). The year 2020 had the highest number of included articles, while 2015 had the lowest (Figure 1).

### 3.1. Quality Assessment

The methodological quality of the included studies was assessed using the JBI critical appraisal checklist. The mean quality score was 5.69 out of 8. Overall, 18 studies (8.3%) were classified as low risk of bias, 180 studies (82.9%) as moderate risk of bias, and 19 studies (8.8%) as high risk of bias. Detailed study‐level quality assessment results are presented in Supporting Table S2.

### 3.2. Pooled Estimation of Resistance Rates

The pooled prevalence of MRSA and the pooled resistance rates to seven commonly reported antibiotics among *Staphylococcus aureus* isolates are presented in Table 1. Regarding MRSA prevalence, the lowest estimate was reported by Oguzkaya‐Artan et al. in Turkey (1.2%), while resistance rates up to 100% were reported by Tayebi et al. (Iran), Aziz et al. (Iraq), Amini et al. (Iran), Hanon et al. (Iraq), and Ismael et al. (Iraq) [12–17]. Regarding vancomycin, resistance rates ranged from 0.0% in many studies to 100% (Ismael et al., Iraq) [15].

**TABLE 1:** Pooled estimates of MRSA prevalence and antibiotic resistance rates among *Staphylococcus aureus* isolates.

Outcome	Overall		
	*N*	Pooled estimation (CI 95%)	Heterogeneity index
MRSA	148	48.60 (44.59–52.60)	I‐squared: 97.39%; *p* < 0.001
Vancomycin	157	2.67 (1.53–4.05)	I‐squared: 96.56%; *p* < 0.001
Gentamicin	145	28.53 (24.45–32.78)	I‐squared: 97.87%; *p* < 0.001
Erythromycin	145	48.92 (45.32–52.53)	I‐squared: 96.53%; *p* < 0.001
Clindamycin	153	34.51 (30.61–38.51)	I‐squared: 97.36%; *p* < 0.001
Ciprofloxacin	150	34.01 (29.80–38.34)	I‐squared: 97.91%; *p* < 0.001
TMP/SMX	143	26.42 (22.00–31.08)	I‐squared: 98.25%; *p* < 0.001
Penicillin G	104	92.32 (89.73–94.61)	I‐squared: 96.21%; *p* < 0.001

*Note:* N: number of studies.

Abbreviation: CI, confidence interval.

**FIGURE 2:**
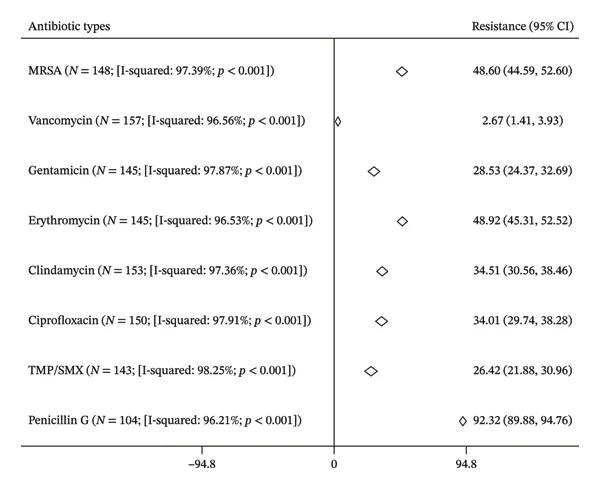
Pooled estimation of resistance rate with 95% confidence interval (CI) separated by antibiotic types. The diamond mark shows the pooled estimation, and the length of the diamond indicates the 95% CI.

As shown in Table 1, considerable heterogeneity was detected among studies for each antibiotic, with *I* ^2^ values exceeding 95% (*p* < 0.001). Therefore, a random‐effects model was applied to estimate pooled resistance rates. The highest pooled resistance rates observed were for penicillin G at 92.32% (95% CI: 89.73–94.61) [*N* = 104; *I* ^2^ = 96.21%; *p* < 0.001] and the lowest for vancomycin at 2.67% (95% CI: 1.53–4.05) [*N* = 157; *I* ^2^ = 96.56%; *p* < 0.001]. Other pooled estimates included MRSA at 48.60% (95% CI: 44.59–52.60), gentamicin 28.53% (95% CI: 24.45–32.78), erythromycin 48.92% (95% CI: 45.32–52.53), clindamycin 34.51% (95% CI: 30.61–38.51), ciprofloxacin 34.01% (95% CI: 29.80–38.34), and TMP/SMX 26.42% (95% CI: 22.00–31.08), all with significant heterogeneity (*p* < 0.001) (Table 1) (Figure 2)

### 3.3. Country‐Wise Pooled Resistance Rates

Overall, marked geographical variation in antimicrobial resistance patterns was observed across the Middle East. Iraq generally exhibited the highest pooled prevalence of MRSA and vancomycin, whereas Yemen showed the highest resistance rates for gentamicin, ciprofloxacin, and TMP/SMX. Turkey consistently demonstrated among the lowest resistance rates for MRSA and vancomycin. Penicillin G resistance remained uniformly high across nearly all countries included in the analysis, highlighting the widespread loss of effectiveness of this antibiotic against *Staphylococcus aureus* in the region. Table 2 presents pooled antibiotic resistance rates by country. In Iran, the highest and lowest pooled resistance rates were for penicillin G (90.76%, 95% CI: 86.56–94.30) [*N* = 52; *I* ^2^ = 96.58%; *p* < 0.001] and vancomycin (1.21%, 95% CI: 0.21–2.73) [*N* = 83; *I* ^2^ = 95.61%; *p* < 0.001], respectively. The highest pooled prevalence of MRSA (70.19%, 95% CI: 44.35–90.70) [*N* = 4; *I* ^2^ = 98.10%; *p* < 0.001] and vancomycin (19.26%, 95% CI: 6.04–37.11) [*N* = 16; *I* ^2^ = 97.70%; *p* < 0.001] were observed in Iraq. The lowest pooled prevalence of MRSA (22.78%, 95% CI: 13.92–33.00) [*N* = 10; *I* ^2^ = 97.05%; *p* < 0.001] and vancomycin (0.0%) were seen in Turkey. The pooled resistance rate of gentamicin was highest in Yemen (76.22%, 95% CI: 62.46–87.84) [*N* = 2] and lowest in Cyprus and Kuwait. The highest pooled resistance rates for gentamicin (76.22%), Cotrimoxazole (73.71%), and ciprofloxacin (71.43%) were all observed in Yemen [*N* = 2 for gentamicin and cotrimoxazole and *N* = 1 for ciprofloxacin], as detailed in Table 2.

**TABLE 2:** Country‐specific pooled estimates of MRSA prevalence and antibiotic resistance rates among *Staphylococcus aureus* isolates.

		Iran	Afghanistan	Cyprus	Egypt	Iraq	Israel	Jordan	Kuwait	Lebanon	Oman	Pakistan	Saudi Arabia	Turkey	Yemen
MRSA	*N*	86	3	1	12	10	4	5	2	1	1	No data	13	10	No data
	Heterogeneity index	*I* ^2^:95.21%; *p* < 0.001	*I* ^2^: 95.83%; *p* < 0.001	—	*I* ^2^:96.16%; *p* < 0.001	*I* ^2^:98.10%; *p* < 0.001	*I* ^2^:98.89%; *p* < 0.001	*I* ^2^:80.18%; *p* < 0.001	—	—	—	—	*I* ^2^:99.09%; *p* < 0.001	*I* ^2^:97.05%; *p* < 0.001	—
	Pooled prevalence	49.89 (45.16–54.62)	43.84 (15.95–74.00)	40.53 (35.96–45.24)	59.22 (43.41–74.16)	70.19 (44.35–90.70)	41.16 (10.90–75.70)	47.92 (35.99–59.97)	55.09 (50.99–59.15)	34.85 (23.53–47.58)	23.81 (8.22–47.17)	—	42.72 (27.96–58.15)	22.78 (13.92–33.00)	—

Vancomycin	*N*	83	2	1	15	16	4	5	2	1	1	1	18	8	No data
	Heterogeneity index	*I* ^2^: 95.61%; *p* < 0.001	*I* ^2^: ‐‐‐; *p* = ‐‐‐	*I* ^2^: ‐‐‐; *p* = ‐‐‐	*I* ^2^: 97.74%; *p* < 0.001	*I* ^2^:97.70%; *p* < 0.001	*I* ^2^:89.24%; *p* < 0.001	*I* ^2^: 0.00%; *p* = 0.97	*I* ^2^: ‐‐‐; *p* = ‐‐‐	*I* ^2^: ‐‐‐; *p* = ‐‐‐	*I* ^2^: ‐‐‐; *p* = ‐‐‐	*I* ^2^: ‐‐‐; *p* = ‐‐‐	*I* ^2^: 90.53%; *p* < 0.001	*I* ^2^: 0.00%; *p* = ‐‐‐	—
	Pooled prevalence	1.21 (0.21–2.73)	0.00 (0.00–0.95)	1.11 (0.36–2.58)	12.77 (1.73–30.20)	19.26 (6.04–37.11)	9.48 (0.28–25.58)	0.00 (0.00–0.45)	0.00 (0.00–0.26)	3.03 (0.37–10.52)	0.00 (0.00–16.11)	0.00 (0.00–7.25)	0.83 (0.06–2.14)	0.00 (0.00–0.00)	—

Gentamicin	N	75	3	1	11	14	3	6	1	1	—	1	19	8	2
	Heterogeneity index	*I* ^2^: 97.51%; *p* < 0.001	*I* ^2^: ‐‐‐; *p* = ‐‐‐	*I* ^2^: ‐‐‐; *p* = ‐‐‐	*I* ^2^: 94.55%; *p* < 0.001	*I* ^2^: 96.28%; *p* < 0.001	*I* ^2^: ‐‐‐; *p* = ‐‐‐	*I* ^2^: 94.28%; *p* < 0.001	*I* ^2^: ‐‐‐; *p* = ‐‐‐	*I* ^2^: ‐‐‐; *p* = ‐‐‐	—	*I* ^2^: ‐‐‐; *p* = ‐‐‐	*I* ^2^: 98.48%; *p* < 0.001	*I* ^2^: 93.71%; *p* < 0.001	*I* ^2^: ‐‐‐; *p* = ‐‐‐
	Pooled prevalence	26.82 (21.25–32.77)	32.27 (0.85–79.32)	0.00 (0.00–0.82)	24.49 (10.70–41.35)	26.33 (10.72–45.52)	6.36 (0.00–26.59)	35.74 (16.28–57.85)	3.39 (2.02–5.08)	50.00 (37.43–62.57)	—	6.12 (1.28–16.87)	32.09 (22.96–41.95)	56.66 (43.68–69.21)	76.22 (62.46–87.84)

Erythromycin	N	70	3	1	12	16	3	6	2	1	1	1	18	10	1
	Heterogeneity index	*I* ^2^: 97.09%; *p* < 0.001	*I* ^2^: ‐‐‐; *p* = ‐‐‐	*I* ^2^: ‐‐‐; *p* = ‐‐‐	*I* ^2^: 89.20%; *p* < 0.001	*I* ^2^: 95.75%; *p* < 0.001	*I* ^2^: ‐‐‐; *p* = ‐‐‐	*I* ^2^: 84.46%; *p* < 0.001	*I* ^2^: ‐‐‐; *p* = ‐‐‐	*I* ^2^: ‐‐‐; *p* = ‐‐‐	*I* ^2^: ‐‐‐; *p* = ‐‐‐	*I* ^2^: ‐‐‐; *p* = ‐‐‐	*I* ^2^: 87.64%; *p* < 0.001	*I* ^2^: 98.88%; *p* < 0.001	*I* ^2^: ‐‐‐; *p* = ‐‐‐
	Pooled prevalence	48.11 (41.82–54.42)	66.57 (52.06–79.67)	26.73 (22.69–31.08)	39.16 (27.31–51.64)	52.39 (36.01–68.53)	55.51 (42.32–68.33)	46.39 (32.30–60.77)	60.95 (56.91–64.92)	81.82 (70.39–90.24)	38.10 (18.11–61.56)	24.49 (13.34–38.87)	48.97 (44.40–53.55)	51.82 (33.53–69.86)	76.19 (52.83–91.78)

Clindamycin	N	74	3	—	15	18	5	7	2	1	1	1	17	8	1
	Heterogeneity index	*I* ^2^:96.83%; *p* < 0.001	*I* ^2^: ‐‐‐; *p* = ‐‐‐	—	*I* ^2^:92.64%; *p* < 0.001	*I* ^2^:93.81%; *p* < 0.001	*I* ^2^:97.79%; *p* < 0.001	*I* ^2^:84.61%; *p* < 0.001	*I* ^2^:‐‐‐; *p* = ‐	*I* ^2^: ‐‐‐; *p* = ‐‐	*I* ^2^: ‐‐; *p* = ‐	*I* ^2^: ‐‐‐; *p* = ‐‐	*I* ^2^:95.00%; *p* < 0.001	*I* ^2^:97.66%; *p* < 0.001	*I* ^2^: ‐‐‐; *p* = ‐‐‐
	Pooled prevalence	32.54 (27.03–38.29)	27.59 (3.25–63.03)	—	29.57 (19.61–40.54)	35.69 (24.20–48.02)	25.97 (5.46–53.40)	32.06 (20.42–44.88)	90.79 (88.26–93.05)	50.00 (37.43–62.57)	38.10 (18.11–61.56)	22.45 (11.77–36.62)	39.19 (32.14–46.46)	43.26 (27.74–59.46)	61.90 (38.44–81.89)

Ciprofloxacin	N	78	3	1	12	19	3	6	1	—	—	1	16	9	1
	Heterogeneity index	*I* ^2^:97.97%; *p* < 0.001	*I* ^2^: ‐‐‐; *p* = ‐‐‐	*I* ^2^: ‐‐‐;*p* = ‐‐‐	*I* ^2^: 96.54%; *p* < 0.001	*I* ^2^: 92.80%; *p* < 0.001	*I* ^2^: ‐‐‐;*p* = ‐‐‐	*I* ^2^: 93.00%; *p* < 0.001	*I* ^2^: ‐‐‐;*p* = ‐‐‐	—	—	*I* ^2^: ‐‐‐;*p* = ‐‐‐	*I* ^2^: 98.29%; *p* < 0.001	*I* ^2^: 99.13%; *p* < 0.001	*I* ^2^: ‐‐‐;*p* = ‐‐‐
	Pooled prevalence	33.22 (26.85–39.90)	48.34 (16.63–80.80)	0.89 (0.24–2.27)	36.05 (20.83–52.79)	30.86 (20.81–41.84)	20.30 (5.11–41.80)	37.12 (19.85–56.19)	57.02 (52.29–61.65)	—	—	6.12 (1.28–16.87)	36.57 (27.34–46.31)	42.02 (15.05–71.76)	71.43 (47.82–88.72)

TMP/SMX	N	75	2	1	13	16	4	5	2	1	1	—	14	8	2
	Heterogeneity index	*I* ^2^:98.44%; *p* < 0.001	*I* ^2^:‐‐‐; *p* = ‐‐‐	*I* ^2^:‐‐‐; *p* = ‐‐‐	*I* ^2^:98.20%; *p* < 0.001	*I* ^2^:86.92%; *p* < 0.001	*I* ^2^:98.01%; *p* < 0.001	*I* ^2^:93.00%; *p* < 0.001	*I* ^2^:‐‐‐; *p* = ‐‐‐	*I* ^2^:‐‐‐; *p* = ‐‐‐	*I* ^2^:‐‐‐; *p* = ‐‐‐	—	*I* ^2^:98.63%; *p* < 0.001	*I* ^2^:89.66%; *p* < 0.001	*I* ^2^:‐‐‐; *p* = ‐‐‐
	Pooled prevalence	25.47 (18.50–33.10)	35.78 (28.17–43.75)	59.91 (55.21–64.48)	38.07 (16.74–61.99)	21.00 (13.32–29.76)	30.27 (5.86–61.85)	20.59 (6.37–39.75)	41.56 (37.54–45.63)	6.06 (1.68–14.80)	4.76 (0.12–23.82)	—	19.21 (10.89–29.13)	27.37 (18.98–36.61)	73.71 (59.66–85.82)

Penicillin G	N	52	3	—	13	9	2	5	—	1	—	1	12	5	2
	Heterogeneity index	*I* ^2^:96.58%; *p* < 0.001	*I* ^2^:‐‐‐; *p* = ‐‐‐	—	*I* ^2^:91.64%; *p* < 0.001	*I* ^2^:59.24%; *p* = 0.001	*I* ^2^:‐‐‐; *p* = ‐‐‐	*I* ^2^: 97.00%; *p* < 0.001	—	*I* ^2^:‐‐‐; *p* = ‐‐‐	—	—	*I* ^2^:85.46%; *p* < 0.001	*I* ^2^:99.46%; *p* < 0.001	*I* ^2^:‐‐‐; *p* = ‐‐‐
	Pooled prevalence	90.76 (86.56–94.30)	95.84 (87.05–99.99)	—	96.01 (90.01–99.61)	94.66 (89.51–98.38)	100 (99.85–100.00)	73.09 (37.16–97.54)	—	84.85 (73.90–92.49)	—	97.96 (89.15–99.95)	96.49 (93.77–98.53)	90.04 (55.42–100.00)	89.14 (77.88–97.10)

### 3.4. Comparative Resistance Rates by Antibiotic Class and Country

In order to depict a more comprehensive resistance trends, the following section summarizes the resistance data by country and the antibiotic type.

#### 3.4.1. MRSA Prevalence

The highest MRSA resistance was observed in Iraq (70.19%; 95% CI: 44.35–90.70), followed by Egypt (59.22%), Kuwait (55.09%), Iran (49.89%), and Jordan (47.92%). The lowest rates were reported in Turkey (22.78%) and Oman (23.81%).

#### 3.4.2. Vancomycin

Resistance to vancomycin was generally low all across the Middle East. The highest prevalence was seen in Iraq (19.26%), Egypt (12.77%), and Israel (9.48%), while several countries including Jordan, Lebanon, Turkey, and Afghanistan exhibited nearly zero resistance.

#### 3.4.3. Gentamicin

The highest resistance rates were recorded in Yemen (76.22%) and Turkey (56.66%). Intermediate levels were seen in Iran (26.82%), Iraq (26.33%), and Egypt (24.49%). The lowest rates were found to be from Kuwait (3.39%), Pakistan (6.12%), and Israel (6.36%).

#### 3.4.4. Erythromycin

Erythromycin resistance was high in almost all countries. Lebanon was found to have the highest rate (81.82%), followed by Yemen (76.19%), Afghanistan (66.57%), Kuwait (60.96%), Israel (55.51%), Iraq (52.39%), Turkey (51.82%), and Iran (48.11%). Cyprus showed the lowest prevalence rate (26.73%).

#### 3.4.5. Clindamycin

Kuwait exhibited a remarkably high resistance rate (90.79%), followed by Yemen (61.90%) and Lebanon (50.00%). Iran (32.54%), Jordan (32.06%), Iraq (35.69%), and Turkey (43.26%) exhibited moderate levels of resistance.

#### 3.4.6. Ciprofloxacin

The highest resistance to ciprofloxacin was found in Yemen (71.43%), Kuwait (57.02%), and Afghanistan (48.34%). Iran (33.22%), Iraq (30.86%), and Turkey (42.02%) fell within the intermediate range, while Cyprus reported the lowest rate (0.89%).

#### 3.4.7. TMP/SMX

Yemen (73.71%) and Cyprus (59.91%) had the highest TMP/SMX resistance. Moderate levels were observed in Iran (25.47%), Iraq (21.00%), Turkey (27.37%), and Egypt (38.07%). The lowest prevalence was found in Oman (4.76%) and Lebanon (6.06%).

#### 3.4.8. Penicillin G

Resistance to penicillin G was high all across the Middle East. Israel reported 100% resistance, followed by Pakistan (97.96%), Saudi Arabia (96.49%), Egypt (96.01%), Iraq (94.66%), and Iran (90.76%). The lowest rate was seen in Jordan (73.09%)

### 3.5. Heterogeneity and Meta‐Regression Analysis

**FIGURE 3:**
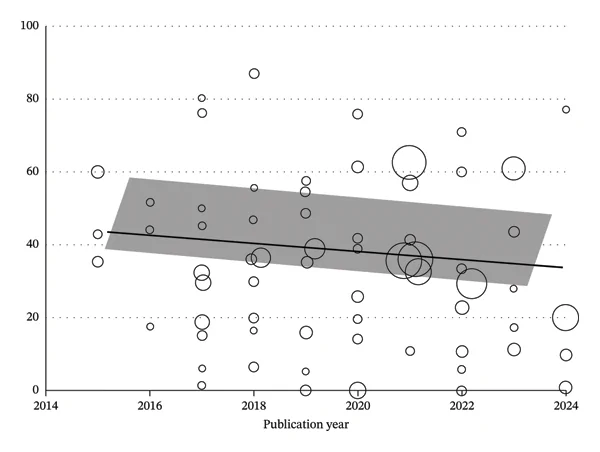
Resistance rate to antibiotic during the study period. Funnel plots are presented in Supporting Figures S1–S8.

Table 1 illustrates a high level of heterogeneity across the studies included in our analysis. Cochran’s Q test was statistically significant (*p* < 0.001), suggesting that this variation is not merely due to chance alone. In fact, the *I* ^2^ statistic exceeded 95% for pooled resistance rates by antibiotic type, implying that more than 95% of observed variance stems from real differences between studies, rather than from sampling error. Univariate meta‐regression analyses were performed to investigate the potential effects of publication year and sample size on the reported resistance rates (Table 3). Publication year was significantly associated with ciprofloxacin resistance (coefficient = −1.67 × 10^−2^; *p* = 0.048), suggesting a decreasing trend in resistance over time (Figure 3). No significant association was observed between sample size and resistance rates for any of the investigated antibiotics. Country‐level differences in resistance patterns were assessed through subgroup analyses (Table 2) rather than meta‐regression, as country represents a categorical variable. For the remaining antibiotics, no statistically significant association was observed between resistance rates and publication year. Further details are presented in Table 3 (Figure 3)

**TABLE 3:** Results of univariate meta‐regression analyses exploring potential sources of heterogeneity in reported resistance rates.

Antibiotics	Publication year (year)		Sample size (number)	
	Coefficient (CI 95%)	*p* value	Coefficient (CI 95%)	*p* value
MRSA	1.42 × 10 ^−2^ (−1.89 × 10^−3^ to 3.02 × 10^−2^)	0.083	−3.39 × 10^−5^ (−1.08 × 10^−4^ to 4.02 × 10^−5^)	0.367
Vancomycin	4.12 × 10^−3^ (−6.16 × 10^−3^ to 1.44 × 10^−2^)	0.430	−9.88 × 10^−6^ (−4.84 × 10^−5^ to 2.86 × 10^−5^)	0.613
Gentamicin	3.50 × 10^−5^ (−1.60 × 10^−2^ to 1.61 × 10^−2^)	0.997	2.09 × 10^−5^ (−5.75 × 10^−5^ to 9.93 × 10^−5^)	0.599
Erythromycin	−9.38 × 10^−3^ (−2.49 × 10^−2^ to 6.16 × 10^−3^)	0.235	1.80 × 10^−6^ (−6.49 × 10^−5^ to 6.85 × 10^−5^)	0.957
Clindamycin	−6.13 × 10^−3^ (−2.20 × 10^−2^ to 9.74 × 10^−3^)	0.447	−6.55 × 10^−7^ (−6.78 × 10^−5^ to 6.65 × 10^−5^)	0.985
Ciprofloxacin	−1.67 × 10^−2^ (−3.32 × 10^−2^ to −1.27 × 10^−4^)	0.048^∗^	1.99 × 10^−6^ (−7.59 × 10^−5^ to 7.99 × 10^−5^)	0.960
TMP/SMX	5.62 × 10^−3^ (−1.15 × 10^−2^ to 2.27 × 10^−2^)	0.516	−2.92 × 10^−5^ (−1.10 × 10^−4^ to 5.12 × 10^−5^)	0.474
Penicillin G	1.01 × 10^−2^ (−4.37 × 10^−3^ to 2.46 × 10^−2^)	0.169	3.24 × 10^−5^ (−4.86 × 10^−5^ to 1.13 × 10^−4^)	0.429

### 3.6. Publication Bias Assessment

Publication bias was evaluated using funnel plots and Egger’s regression test. Visual inspection of funnel plots revealed varying degrees of asymmetry across several outcomes. Egger’s test suggested potential small‐study effects for MRSA, gentamicin, erythromycin, clindamycin, TMP/SMX, and penicillin G. In contrast, no significant asymmetry was observed for vancomycin resistance. Given the substantial between‐study heterogeneity (*I* ^2^ > 95% for most analyses), these findings should be interpreted with caution, as funnel plot asymmetry may reflect genuine heterogeneity and methodological differences rather than publication bias alone. Funnel plots are presented in Supporting Figures S1–S8.

### 3.7. Sensitivity Analysis

Leave‐one‐out sensitivity analyses were performed for all outcomes. Sequential omission of individual studies did not result in substantial changes in the pooled resistance estimates for MRSA, vancomycin, gentamicin, erythromycin, clindamycin, ciprofloxacin, TMP/SMX, or penicillin G. The direction and magnitude of the pooled estimates remained generally stable throughout the analyses, indicating that no single study exerted a disproportionate influence on the overall results. These findings support the robustness of the pooled estimates despite the high heterogeneity observed across studies.

## 4. Discussion

This systematic review and meta‐analysis aimed to assess the antibiotic resistance patterns of *Staphylococcus aureus* across Middle Eastern countries. To our knowledge, this meta‐analysis is the first comprehensive regional synthesis of *Staphylococcus aureus* resistance patterns across Middle Eastern countries. The findings revealed alarmingly high pooled resistance rates, especially for penicillin G (92.32%), erythromycin (48.92%), and MRSA (48.60%). In contrast, resistance to vancomycin remains relatively low (2.67%), which means that it is still a viable treatment option, if indicated. Nevertheless, the pooled prevalence of vancomycin resistance observed in this study should be interpreted with caution. Reported resistance rates may have been influenced by methodological differences across studies, including variability in susceptibility testing methods, interpretive criteria (e.g., CLSI versus EUCAST breakpoints), and the potential inclusion of VISA isolates in some reports. Furthermore, vancomycin susceptibility testing in *S. aureus* is known to be method‐dependent, and certain methods may overestimate resistance compared with reference MIC‐based techniques. These factors may partially explain the variation in reported vancomycin resistance rates across the included studies. Considerable heterogeneity was observed across studies and countries, highlighting variations in antibiotic use and potential differences in national antimicrobial stewardship practices. Iraq had the highest pooled resistance rates for both MRSA and vancomycin, followed by Egypt. The lowest pooled resistance rates for MRSA and gentamicin were both observed in Turkey. However, Yemen showed the highest pooled resistance rates to ciprofloxacin, gentamicin, and cotrimoxazole; however, these estimates were based on a limited number of studies and should therefore be interpreted cautiously. The resistance rates observed in the present study are broadly consistent with global concerns regarding the increasing burden of antimicrobial resistance in *Staphylococcus aureus*. Similar trends of high resistance to penicillin and macrolides have been reported in surveillance studies from Europe, Asia, and Africa. However, substantial geographical variation exists worldwide, reflecting differences in antibiotic stewardship programs, healthcare infrastructure, prescribing practices, infection control measures, and regulatory policies governing antimicrobial use. These international observations support the marked intercountry variability identified across the Middle East in the current analysis [6, 8, 18, 19].

### 4.1. Factors Contributing to Resistance

#### 4.1.1. Ineffective Antibiotic Stewardship and Self‐Medication

One of the main factors attributed to increasing antibiotic resistance in the Middle East is incorrect patient behavior toward consumption of these medications. In this section, we review the studies that have reported or assessed misuse and self‐medication behaviors in different countries across the Middle East. Antibiotic self‐medication might be one of the contributing factors to the growing antibiotic resistance issue globally. This group of medications is the most common drugs stored and used at home [20]. Studies revealed that people commonly obtain antibiotics from pharmacies without prescriptions and store leftover drugs for use by friends/relatives. Antibiotics are also the most commonly reported prescription drugs with high suspicion of abuse/misuse [21]. Antibiotics are the second most common medicines that students consume without prescription in Turkey [22]. The act of self‐medications was prevalent among 92.4% of college students in Iraq, 63.4% in Turkey, and 77.7% in Egypt [22–24]. Self‐prescription percentages among antibiotics group include penicillin, cephalosporins, erythromycin, clindamycin, aminoglycosides, and co‐trimoxazole, which were reported to be 78.29%, 77.36%, 64.39%, 27.77%, 35.24%, and 74.86%, respectively [20]. Household storage of medicines may also play a role in the irrational use of antibiotics. About 44.7% of those who use prescribed or nonprescribed antibiotics reported that they kept left‐over antibiotics from the incomplete treatment course for future need. The availability of antibiotics without prescription is found to be positively associated with self‐medication [25]. In addition to general evidence of self‐medication, drawing analogies between such behaviors and resistance patterns observed in certain countries may help in understanding the relationship between such behaviors and antibiotic resistance. The highest pooled resistance rates for gentamicin (76.22%), cotrimoxazole (73.71%), and ciprofloxacin (71.43%) were all observed in Yemen, where the sale of antibiotic without prescription is widespread, particularly for the treatment of sore throat and cough [26]. A study done by Belkina et al. showed that pharmacies were the main source of nonprescribed antibiotics in both Yemen and Saudi Arabia [27]. In addition to patient self‐prescription, the dispensing of antibiotics by pharmacists without a prescription is a potential factor contributing to high resistance rates in Iraq. Several studies have reported that community pharmacists frequently dispense antibiotics without prescriptions, underscoring the widespread self‐medication and misuse of these drugs in the country [28]. Both self‐prescription and irrational antibiotic dispensing by pharmacists have been documented in several countries, including Egypt, and these practices may contribute to high resistance rates. In our study, we observed that Egypt had the second highest pooled prevalence of vancomycin‐resistant (12.77%) and MRSA (59.22%). Similar to Iraq, numerous studies report widespread self‐medication and pharmacist dispensing without prescription in Egypt. In many Middle Eastern countries, including Egypt, the absence of strict antibiotic dispensing regulations in community pharmacies facilitates this misuse. For example, amoxicillin is frequently dispensed without pharmacists collecting adequate patient information, reflecting systemic weakness in antibiotic stewardship [29]. On the other hand, countries with stricter stewardship of nonprescription antibiotic sales demonstrate markedly lower resistance rates. In our study, the lowest pooled resistance to methicillin and gentamicin was observed in Turkey, where selling antibiotics without a prescription is illegal. Turkish pharmacists reported that they never dispense antibiotics without a prescription and noted that patients’ requests for such medications have decreased in recent years, although demands—particularly for respiratory tract infections—still occur repeatedly [30]. In Jordan, beta‐lactam antibiotics are the most commonly dispensed group in pharmacies, both with and without a prescription. There is also a tendency to use higher‐generation antibiotics for cases that could be effectively managed with lower‐generation or safer alternatives. Furthermore, 12.2% of antibiotics were dispensed for infections that were not indicated for. These results suggest that prescription errors by physicians, in addition to nonprescription dispensing by pharmacist, play a significant role in antibiotic misuse.

#### 4.1.2. Prescription Errors and Inappropriate Dosing

Apart from self‐prescription and nonprescription dispensing, prescribing errors and inappropriate dosing significantly contribute to antibiotic resistance patterns observed across the Middle East. In the Gulf region, 43.3% of antibiotic prescribing were deemed inappropriateness [31]. Most physicians in public hospitals prescribed antibiotics empirically, without relying on culture and sensitivity tests [28], and 74% of prescribers in Yemen reported pressure to prescribe broad‐spectrum agents. Antimicrobial sensitivity testing was often omitted due to high costs [32]. In Oman, the overall dosing inappropriateness was reported as 29.5%, with vancomycin being the most common parenteral antibiotic affected (19.8%), while trimethoprim–sulfamethoxazole was the most inappropriately prescribed oral agent (28.6%) [33]. Similarly, inappropriate vancomycin indications and dosing regimens were reported in Iran and Saudi Arabia [34, 35]. More than two‐thirds of the antimicrobials in hospitals across the region are prescribed empirically, with point prevalence studies showing high rates of inappropriate antimicrobial use in Turkish hospitals [36]. In intensive care units, inappropriate antibiotic use against Gram‐positive bacteria was reported as high as 83% [37]. Likewise, a study done in Pakistan found that approximately two‐thirds of patients received at least one inappropriate antimicrobial [38]. Collectively, these findings highlight the urgent need to strengthen diagnostic stewardship, improve prescribing accuracy, and ensure appropriate dosing as core components of antibiotic resistance mitigation strategies.

#### 4.1.3. Antibiotic Prescription Trends

Variations in resistance rates observed in this study may be partly explained by differences in prescription practices across countries. In Iran, penicillin G pooled resistance in Iran was 90.76%, consistent with studies showing penicillin as one of the most commonly used outpatient antibiotics [39]. In Egypt, penicillin G pooled resistance in Egypt was even higher at 96.01%. Research on Egyptian community pharmacies found that penicillins and quinolones were the two major classes dispensed on prescription, while first‐generation cephalosporins and quinolones were most frequently recommended by pharmacists; penicillins and macrolides were most often dispensed at patients’ request [40]. The high frequency of penicillin use may explain why resistance to this drug was the highest among all subject antibiotics in our study. By contrast, Jordan had the lowest pooled resistance to penicillin G (73.09%). A study by Al‐Niemat reported that macrolides (primarily azithromycin) and amoxicillin/clavulanate were the most commonly prescribed antibiotic (40% and 25%, respectively), while penicillin accounted for only 21% [41]. This comparatively lower prescribing frequency may help explain Jordan’s reduced resistance levels. In Yemen, however, amoxicillin, trimethoprim–sulfamethoxazole, and amoxicillin/clavulanic acid accounted for 85% of prescriptions [42]. The heavy use of cotrimoxazole is consistent with the high resistance level to this antibiotic observed in our study. In Saudi Arabia, co‐amoxiclav and fluoroquinolones are the most frequently prescribed antibiotics in the outpatient and emergency departments [43]. Correspondingly, ciprofloxacin resistance in *Staphylococcus aureus* was 36.57%, relatively modest compared to other countries. Yemen, by contrast, showed the highest gentamicin resistance (76.22%) and the second‐highest erythromycin resistance (76.19%). Macrolides were the most frequently prescribed class by Yemeni dentists (72%), with azithromycin (18%), erythromycin (15%), and clarithromycin (10%) being the most common, while aminoglycosides were less common (21%), with gentamicin at only 8% [44]. These findings suggest that excessive macrolide and gentamicin use in dentistry may be a key driver of Yemen’s high resistance rates. Iran demonstrated comparatively lower pooled resistance rates for erythromycin (48.11%) and gentamicin (26.82%). Gentamicin accounted for less than 1.1% of prescriptions and erythromycin for 1.2% among Iranian dentists [45]. Similarly, survey data from Iraq and Egypt revealed extremely low prescription rates for erythromycin and gentamicin, at 0.2% and 0.4%, respectively [46, 47]. These stark differences highlight how Yemen’s dental prescribing practices, compared with other Middle Eastern countries, likely contribute to disproportionately high resistance levels. Overall, the evidence underscores the urgent need for standardized and controlled prescribing guidelines across the region.

#### 4.1.4. Excessive Antibiotic Use in Agriculture

The extensive and often uncontrolled use of antibiotics in livestock and poultry production, alongside their use in human medicine, contributes significantly to the emergence and spread of resistant strains in the environment. In poultry farming, antibiotics are commonly added to feed and water at subtherapeutic levels for growth promotion and prophylaxis, resulting in unnecessary exposure of even healthy birds. Competition for food sources leads to variations in individual intake, imposing differential selective pressures on commensal bacteria and promoting the emergence of resistant strains that may ultimately contaminate the environment [48, 49]. In Egypt, antibiotic residues were detected in 23% of chicken meat samples for oxytetracycline (OTC), 21% for gentamicin, and 17% for ciprofloxacin, although boiling for 30 min significantly reduced residue levels [50]. In Iran, 50% of poultry muscle samples and 71.42% of liver samples tested positive for gentamicin residues, both exceeding standard limit [51]. These findings highlight the urgent need for stricter regulations governing antibiotic use in food‐producing animals, alongside robust medical stewardship, to prevent resistance from spreading across species and into the environment. The interpretation of pooled resistance estimates should also consider the substantial between‐study heterogeneity observed across analyses. Differences in study design, patient populations, laboratory methods, and reporting practices may have influenced the observed estimates and contributed to funnel plot asymmetry in some outcomes. It should be noted that the present meta‐analysis was not designed to directly evaluate the effects of self‐medication, prescribing practices, antimicrobial stewardship, or agricultural antibiotic use on resistance rates. Therefore, the factors discussed above should be interpreted as potential contributors identified from the broader literature rather than as causal determinants of the resistance patterns observed in this study. Future analytical studies are needed to directly examine these relationships. Taken together, these findings highlight the multifactorial nature of antimicrobial resistance in the Middle East, with potentially modifiable factors operating at the patient, prescribing, healthcare, agricultural, and surveillance levels. These factors are summarized in Table 4.

**TABLE 4:** Major factors contributing to antimicrobial resistance and potential mitigation strategies.

Factors contributing to antimicrobial resistance	Possible mechanism	Potential interventions
Self‐medication with antibiotics	Inappropriate antibiotic exposure increases selective pressure	Strengthen prescription‐only regulations; public education
Over‐the‐counter antibiotic dispensing	Uncontrolled antibiotic consumption	Enforce pharmacy regulations and antimicrobial stewardship
Inappropriate prescribing and empirical therapy	Unnecessary or broad‐spectrum antibiotic use	Culture‐guided therapy; stewardship programs
Inappropriate dosing or treatment duration	Subtherapeutic exposure promotes resistance	Follow evidence‐based clinical guidelines
Poor infection prevention and control	Transmission of resistant strains	Hand hygiene; infection prevention and control
Excessive antibiotic use in agriculture	Selection of resistant bacteria in animals/environment	Restrict nontherapeutic veterinary antibiotic use
Limited antimicrobial surveillance	Delayed detection of emerging resistance	Strengthen AMR surveillance systems
Nutritional support	Supports host immune function but does not directly reverse resistance	Balanced diet as supportive care alongside antibiotics
Natural bioactive compounds	Experimental antimicrobial activity reported for some compounds	Further clinical research before routine recommendation

*Note:* Dietary measures and natural products should be considered supportive approaches and should not replace evidence‐based antimicrobial therapy.

### 4.2. Limitations

There are a number of limitations in this study. First, our findings may not be as broadly applicable to the entire Middle East due to a limited number of high‐quality studies available in some countries. Second, only a subset of antibiotics could be included in the final meta‐analysis because of the large volume of retrieved data and wide variability in reported antibiotic classes. Despite being methodologically necessary, this restricted inclusion may not capture the full spectrum of resistance pattern across the regions. Finally, differences in sampling strategies and resistance measurement techniques among the included studies may have contributed to heterogeneity in the pooled estimates. In addition, publication bias could not be completely excluded. Although publication bias assessment indicated possible asymmetry for some outcomes, the very high heterogeneity and methodological diversity among studies may have contributed to these findings. Therefore, the results should be interpreted cautiously. In addition, the geographical definition of the Middle East is not universally standardized. The inclusion of Afghanistan and Pakistan reflects their participation in several regional public health and antimicrobial resistance surveillance frameworks; however, their classification may differ across geographical definitions. Furthermore, several country‐specific estimates were derived from only one or two studies. Therefore, these pooled estimates should be interpreted with caution, as they may not fully represent the national resistance patterns and may be more susceptible to random variation. Additional surveillance studies are needed in underrepresented countries to improve the reliability and generalizability of regional comparisons.

## 5. Conclusions

Our findings underscore the urgent need for stricter regulatory frameworks governing the sale and distribution of antibiotics across the Middle East. Enforcing prescription‐only antibiotic dispensing policies in community pharmacies, particularly in countries with high rates of nonprescription access, is critical. Priorities should also include improving antibiotic stewardship programs, promoting evidence‐based prescribing by physicians, and lowering empirical antibiotic use of the availability of affordable diagnostic tools. Public health education campaigns highlighting the dangers of self‐medication and antibiotic misuse are equally important. Lastly, regulations on the nontherapeutic use of antibiotics in livestock and poultry shall be extended, given the significant role of veterinary antibiotic use in driving resistance.

## Author Contributions

Omid Raiesi, Sarina Alidadpour, Mohammad Hossein Ekvan, Gita Alizadeh, Wrya Parwaie, and Muhammad Ibrahim Getso were responsible for data processing, data collection, conducting experiments, drafting the manuscript, creating visualizations, and critically revising or editing the article. Omid Raiesi, Sarina Alidadpour, Gita Alizadeh, Wrya Parwaie, and Reza Pakzad conceived the study and contributed to the study design, analysis, interpretation of results, critical revision or editing of the article, provided the final approval for publication, supervised the project, and acquired funding.

## Funding

This study was funded by the Ilam University of Medical Sciences, Ilam, Iran, under grant number IR.MEDILAM.REC.1403.193.

## Ethics Statement

The Ethical Committee of the Ilam University of Medical Sciences, Ilam, Iran, approved this research with the code IR.MEDILAM.REC.1403.193.

## Consent

The authors have nothing to report.

## Conflicts of Interest

The authors declare no conflicts of interest.

## Supporting Information

Additional supporting information can be found online in the Supporting Information section.

## Supporting information


**Supporting Information** Supporting Table S1 provides the detailed search strategies, including database‐specific search strings and Boolean operators used for the systematic literature search. Supporting Table S2 presents the study‐level quality assessment results. Supporting Figures S1–S8 provide the funnel plots used to assess potential publication bias across the meta‐analyses.

## Data Availability

In this study, all data and materials are included. If more information is needed, please contact the author for data requests.
