# Supplementary material for: Analysis of Antibiotic Resistance Patterns and Distribution of Staphylococcus aureus in the Middle East: A Systematic Review and Meta‐Analysis
Source: Can J Infect Dis Med Microbiol. 2026 Sep 26;2026:2830917. doi: 10.1155/cjid/2830917 (PMC13615457; doi:10.1155/cjid/2830917)
Supplement: Supplementary file 1 [file CJID-2026-2830917-s001.zip › Supplementary Table S1.docx]

**Supplementary Table S1. Database-specific search strategies
PubMed**
(("Staphylococcus aureus"[Mesh] OR "Staphylococcus aureus" OR "S. aureus") AND ("Drug Resistance, Bacterial"[Mesh] OR "Antimicrobial Resistance"[Mesh] OR "antibiotic resistance" OR "antimicrobial resistance" OR "drug resistance" OR "drug susceptibility" OR resistant OR susceptibility) AND (Iran OR Iraq OR Turkey OR Egypt OR Saudi Arabia OR Jordan OR Kuwait OR Lebanon OR Oman OR Yemen OR Israel OR Palestine OR Cyprus OR Bahrain OR Qatar OR "United Arab Emirates" OR Afghanistan OR Pakistan)) AND ("2015/01/01"[Date - Publication] : "2024/09/24"[Date - Publication])
**Web of Science**
TS=("Staphylococcus aureus" OR "S. aureus") AND TS=("antibiotic resistance" OR "antimicrobial resistance" OR "drug resistance" OR "drug susceptibility" OR resistant OR susceptibility) AND TS=(Iran OR Iraq OR Turkey OR Egypt OR "Saudi Arabia" OR Jordan OR Kuwait OR Lebanon OR Oman OR Yemen OR Israel OR Palestine OR Cyprus OR Bahrain OR Qatar OR "United Arab Emirates" OR Afghanistan OR Pakistan)
Timespan: January 2015 – September 24, 2024
**Scopus**
TITLE-ABS-KEY("Staphylococcus aureus" OR "S. aureus") AND TITLE-ABS-KEY("antibiotic resistance" OR "antimicrobial resistance" OR "drug resistance" OR "drug susceptibility" OR resistant OR susceptibility) AND TITLE-ABS-KEY(Iran OR Iraq OR Turkey OR Egypt OR "Saudi Arabia" OR Jordan OR Kuwait OR Lebanon OR Oman OR Yemen OR Israel OR Palestine OR Cyprus OR Bahrain OR Qatar OR "United Arab Emirates" OR Afghanistan OR Pakistan) AND PUBYEAR > 2014
**Google Scholar**
("Staphylococcus aureus" OR "S. aureus") AND ("antibiotic resistance" OR "antimicrobial resistance" OR "drug resistance") AND (Iran OR Iraq OR Turkey OR Egypt OR "Saudi Arabia" OR Jordan OR Kuwait OR Lebanon OR Oman OR Yemen OR Israel OR Palestine OR Cyprus OR Bahrain OR Qatar OR "United Arab Emirates" OR Afghanistan OR Pakistan)
Filters applied: • Publication date: January 2015 – September 24, 2024 • English language • Human studies • Full-text articles available
